# Supplementary material for: Digital Workflow for Interim Prosthetic Rehabilitation Through the All-on-4 Concept Using 3D Printing Additive Process
Source: J Clin Med. 2025 Nov 24;14(23):8353. doi: 10.3390/jcm14238353 (PMC12693154; doi:10.3390/jcm14238353)
Supplement: Supplementary file 1 [file jcm-14-08353-s001.zip › jcm-3985821-supplementary.pdf]

CARE case report guidelines

CARE Checklist of information to include when writing a case report

(cc) BY-NC-ND

Topic	Item	Checklist item description	Reported on Line
Title	1	The diagnosis or intervention of primary focus followed by the words "case report"	
Key Words	2	2 to 5 key words that identify diagnoses or interventions in this case report, including "case report"	
Abstract (no references)	3a	Introduction: What is unique about this case and what does it add to the scientific literature?	Lines 14-20
	3b	Main symptoms and/or important clinical findings	Lines 25-32
	3c	The main diagnoses, therapeutic interventions, and outcomes	Lines 20-25
	3d	Conclusion—What is the main "take-away" lesson(s) from this case?	Lines 32-35
Introduction	4	One or two paragraphs summarizing why this case is unique (**may include references**)	
Patient Information	5a	De-identified patient specific information.	Lines 83-96
	5b	Primary concerns and symptoms of the patient.	Lines 98-102
	5c	Medical, family, and psycho-social history including relevant genetic information	Lines 90-96
	5d	Relevant past interventions with outcomes	
Clinical Findings	6	Describe significant physical examination (PE) and important clinical findings.	
Timeline	7	Historical and current information from this episode of care organized as a timeline	Lines 105-145
Diagnostic Assessment	8a	Diagnostic testing (such as PE, laboratory testing, imaging, surveys).	
	8b	Diagnostic challenges (such as access to testing, financial, or cultural)	
	8c	Diagnosis (including other diagnoses considered)	
	8d	Prognosis (such as staging in oncology) where applicable	
Therapeutic Intervention	9a	Types of therapeutic intervention (such as pharmacologic, surgical, preventive, self-care)	Lines 105-145
	9b	Administration of therapeutic intervention (such as dosage, strength, duration)	Lines 105-117
	9c	Changes in therapeutic intervention (with rationale)	
Follow-up and Outcomes	10a	Clinician and patient-assessed outcomes (if available)	Lines 161-218
	10b	Important follow-up diagnostic and other test results	Lines 234-317
	10c	Intervention adherence and tolerability (How was this assessed?)	Lines 307-317
	10d	Adverse and unanticipated events	Lines 240-242
Discussion	11a	A scientific discussion of the strengths AND limitations associated with this case report	Lines 420-426
	11b	Discussion of the relevant medical literature **with references**.	Lines 320-419
	11c	The scientific rationale for any conclusions (including assessment of possible causes)	Lines 429-435
	11d	The primary "take-away" lessons of this case report (without references) in a one paragraph conclusion	Lines 429-432
Patient Perspective	12	The patient should share their perspective in one to two paragraphs on the treatment(s) they received	Lines 408-419
Informed Consent	13	Did the patient give informed consent? Please provide if requested	Yes ☒ No ☐
